# Histone Deacetylase GiSRT2 Negatively Regulates Flavonoid Biosynthesis in *Glycyrrhiza inflata*

**DOI:** 10.3390/cells12111501

**Published:** 2023-05-29

**Authors:** Jiangyi Zeng, Yun Huang, Lijun Zhou, Xiaoju Liang, Chao Yang, Hongxia Wang, Ling Yuan, Ying Wang, Yongqing Li

**Affiliations:** 1 Guangdong Provincial Key Laboratory of Applied Botany & Guangdong Provincial Key Laboratory of Digital Botanical Garden, South China Botanical Garden, Chinese Academy of Sciences, Guangzhou 510650, China; zengjiangyi@scbg.ac.cn (J.Z.); huangyun18@scbg.ac.cn (Y.H.); 15810928775@163.com (L.Z.); liangxiaoju718@163.com (X.L.);; 2 College of Life Science, Gannan Normal University, Ganzhou 341000, China; 3 University of Chinese Academy of Sciences, Beijing 100049, China; hxwang@cemps.ac.cn; 4 Shanghai Key Laboratory of Plant Functional Genomics and Resources, Shanghai Chenshan Botanical Garden, Shanghai 201602, China; 5 Department of Plant and Soil Sciences, University of Kentucky, Lexington, KY 40506, USA; lyuan3@uky.edu

**Keywords:** histone deacetylase, *O*-methyltransferase, licochalcone A, flavonoid, licorice

## Abstract

*Glycyrrhiza inflata* Batalin is a medicinal licorice species that has been widely used by humans for centuries. Licochalcone A (LCA) is a characteristic flavonoid that accumulates in *G. inflata* roots with high economical value. However, the biosynthetic pathway and regulatory network of its accumulation remain largely unknown. Here we found that a histone deacetylase (HDAC) inhibitor nicotinamide (NIC) could enhance the accumulation of LCA and total flavonoids in *G. inflata* seedlings. *GiSRT2*, a NIC-targeted HDAC was functionally analyzed and its RNAi transgenic hairy roots accumulated much more LCA and total flavonoids than its OE lines and the controls, indicating a negative regulatory role of *GiSRT2* in the accumulation of LCA and total flavonoids. Co-analysis of transcriptome and metabolome of RNAi-*GiSRT2* lines revealed potential mechanisms in this process. An *O*-methyltransferase gene, *GiLMT1* was up-regulated in RNAi-*GiSRT2* lines and the encoded enzyme catalyzed an intermediate step in LCA biosynthesis pathway. Transgenic hairy roots of *GiLMT1* proved that *GiLMT1* is required for LCA accumulation. Together, this work highlights the critical role of *GiSRT2* in the regulation of flavonoid biosynthesis and identifies *GiLMT1* as a candidate gene for the biosynthesis of LCA with synthetic biology approaches.

## 1. Introduction

Licorice is a popular medicinal herb that has been used by humans worldwide for centuries. The main active components of licorice are triterpenoids and flavonoids. Among them, licochalcone A (LCA) is the characteristic flavonoid in *Glycyrrhiza inflata* Batalin [1], one of the major medicinal licorice species. LCA has a wide range of pharmacological properties. To date, it has been discovered that LCA has excellent activity to inhibit the proliferation and motility of 17 classes of cancer cells [2,3]. Furthermore, LCA has demonstrated anti-inflammatory effects against both acute and chronic inflammation [4,5]. Moreover, LCA has been shown to have antioxidant activity and neuroprotective effects [6,7]. LCA is also a valuable compound used in the pharmaceutical and cosmetics industries. However, the limited yield of LCA restricts its application, which motivates research into methods for improving LCA production.

Despite its great commercial value, LCA only accumulates in low abundance in a very limited number of plant species. *G. inflata* is the major resource for LCA on the market. However, it usually takes years before the roots can be harvested for LCA extraction with the yield too low to meet the market demand. The chemical synthesis protocols of LCA have been long reported and optimized, but due to the complexity and high cost of the process, no protocols have been used in industry so far [8]. The biosynthetic pathway of LCA as well as the underlying molecular regulation mechanisms have been rarely reported. Based on the structure of LCA, we hypothesized that P450s, *O*-methyltransferases, reductases, and prenyltransferases should be involved in the biosynthesis of LCA. Identification of these genes could provide candidate genes to reconstruct the LCA biosynthetic pathway in plants or microorganisms to produce LCA in large amounts with synthetic biology approaches. The close structural similarity of echinatin and LCA suggests that echinatin might be the direct precursor of LCA. In previous studies, the first two steps from liquiritigenin to echinatin have been analyzed: flavanone 2-hydroxylase (F2H) converts liquiritigenin to licodione, and licodione 2′-*O*-methyltransferase (LMT) then adds a methyl group to licodione [9,10]. However, there is no evidence to demonstrate that F2H and LMT contribute to the biosynthesis of LCA.

Furthermore, elucidation of the molecular regulatory network of LCA biosynthesis could provide the theoretical basis to improve field cultivation techniques for higher LCA yield. We previously found that overexpression of *AtMYB12* in transgenic hairy roots of *G. inflata* could promote the accumulation of echinatin and LCA by inducing the expression of *GiCHS1*, suggesting that the homologue of *AtMYB12* in *G. inflata* might also possess a similar function [11]. As a specialized metabolite accumulated in licorice roots, LCA is responsive to different environmental cues. Histone deacetylases (HDACs) are important epigenetic regulators that regulate plant responses to environmental factors and the biosynthesis of multiple metabolites by removing acetyl groups from histone or non-histone proteins. For instance, silencing *SlHDT3* in tomato decreased carotenoid accumulation [12]. In *Arachis hypogaea* hairy roots, overexpression of *AhHDA1* promotes the accumulation of flavonoids [13]. Treatment of HDAC inhibitors increases the content of ginsenoside in ginseng adventitious roots by cooperating with MeJA [14].

Sirtuins are members of the NAD-dependent deacetylase family that act on histone and non-histone proteins and have been shown to have different enzymatic activities and subcellular localization. They are reported to regulate plant growth and development and responses to biotic and abiotic stresses [15], as well as energy metabolism, metabolite transport, and ethylene signaling [16,17]. Zhang et al. demonstrated that OsSRT1 represses glycolysis by both regulating epigenetic modification of histone and inhibiting the moonlighting function of GAPDH as a transcriptional activator of glycolytic genes in rice [18].

**Nicotinamide (NIC)** is a general pan-inhibitor of sirtuins [19]. In this study, we found that NIC treatment suppressed root growth of *G. inflata* seedlings with enhanced accumulation of LCA and total flavonoids. We further identified an SIR class HDAC, *GiSRT2*, as a negative regulator of the LCA accumulation process in *G. inflata*. The RNAi-*GiSRT2* transgenic hairy roots accumulated much more LCA and total flavonoids than the OE-*GiSRT2* lines and controls. Combined transcriptomic and metabolomic analysis provided insights into the biosynthesis of LCA and its molecular regulation mechanisms. An *LMT* gene, *GiLMT1*, was up-regulated in RNAi-*GiSRT2* lines. Enzyme assay and transgenic hairy root analysis further confirmed the involvement of GiLMT1 in LCA biosynthesis. Taken together, this study provides not only a theoretical basis for improving the yield of LCA in the field, but also a candidate gene for the biosynthesis of LCA with synthetic biology approaches.

## 2. Materials and Methods

### 2.1. Plant Materials and NIC Treatment

*G. inflata* seeds were provided by Gansu Jin You Kang Pharmaceutical Technology Co., Ltd. The seeds were surface-sterilized and cultured as previously described [11]. Seven-day-old seedlings were treated on 1/2MS plates supplemented with 1 mM NIC. Untreated seedlings were used as controls.

### 2.2. RNA Extraction and qRT-PCR Analysis

RNA extraction and qRT-PCR were carried out as previously described [20]. *GiCOPS3* was used as internal reference gene [21]. All primers used in qRT-PCR are listed in Table S1.

### 2.3. Vector Construction

The genome sequence of *G. inflata* used in this study was provided by our research group (https://ngdc.cncb.ac.cn/; NGDC; CRA009044; accessed on 25 November 2022). The coding sequences (CDS) of *GiSRT2* (accession number OQ982386) and *GiLMT1* (accession number OQ982385) were PCR-amplified from cDNAs, digested with *Sal* I and *Kpn* I, or *Pst* I and *Spe* I enzymes, respectively, and cloned into *pSuper1300-GFP* to generate OE-*GiSRT2* and OE-*GiLMT1* transgenic hairy roots, respectively. The *pRNAiGG* vector [22] was used to generate RNAi-*GiSRT2* and RNAi-*GiLMT1* transgenic hairy roots. The gene-specific fragments were PCR amplified and cloned into the *pRNAiGG* vector using the *Bsa* I restriction site. The CDS of *GiLMT1* was cloned into the *E. coli* expression vector *pColdII* (TaKaRa) using *Sac* I and *Sal* I restriction sites to produce a recombinant protein. The full-length CDS of *GiSRT2* was cloned into *pCAMBIA1300-UBQ-GFP* in frame with GFP for subcellular localization analysis. All vectors were confirmed using DNA sequencing (Qingke, Beijing, China).

### 2.4. Generation of Transgenic Hairy Roots of G. inflata

Transgenic hairy roots were generated as previously described with minor modifications [11]. Binary vectors were introduced into the modified *A. rhizogenes* strain MSU440. Three different types of controls were used in this work, including non-transgenic hairy roots (WT), OE empty vector-induced hairy roots (OE-CK), and RNAi empty vector-induced hairy roots (RNAi-CK). PCR with *rolB* (from Ri plasmid of MSU440) and gene-specific primers was performed to identify the positive transgenic lines. All the primers used in this study are listed in Table S1.

### 2.5. Compound Extraction and Determination

To extract the metabolites, 10 mg of freeze-dried samples was subjected to ultrasonic-assisted extraction twice with 1 mL of methanol for 30 min. After centrifugation at 12,000× *g* for 10 min, the supernatants were dried and finally redissolved with 300 μL of methanol.

Total flavonoids content was measured using the sodium nitrite–aluminum nitrate colorimetric method using rutin as the standard [23].

The contents of specialized metabolites were analyzed using HPLC. The supernatant samples were filtered through 0.22 μm millipore filters. The samples were then analyzed using HPLC (LC-2030C, Shimadzu, Kyoto, Japan), using a reversed-phase C18 column (150 mm × 4.6 mm, 5 μm, Shimadzu, Kyoto, Japan) under the following chromatographic conditions: mobile phase, acetonitrile (A) and 0.1% formic acid (B); flow rate, 1 mL/min; sample injection volume, 10 µL; detection wavelengths, 254 nm and 370 nm; column temperature, 40 °C. The gradient elution approach was set as follows: 0~25 min, 30~55% A; 25~27 min, 55~95% A; 27~30 min, 95% A; 30~31 min, 95~30% A; 31~35 min, 30% A. The standards of rutin, echinatin, isoliquiritigenin, licochalcone C, and LCA were purchased from Biosynth Carbosynth, Staad, Switzerland.

### 2.6. Transcriptomic and Metabolomic Analysis

Hairy roots of RNAi-*GiSRT2* and the corresponding CK lines (RNAi empty vector-induced hairy roots) were sampled for RNA-seq and metabolite analyses by Metware Biotechnology Co., Ltd. (Wuhan, China) as previously described [24]. Differentially expressed genes (DEGs) were selected with |log_2_(Fold Change)| ≥ 1 (|log_2_FC| ≥ 1) and *p*-value < 0.05. Gene Ontology (GO) and Kyoto Encyclopedia of Genes and Genomes (KEGG) enrichment analyses of DEGs were implemented using the OmicShare tools accessed on 9 February 2023 (http://www.omicshare.com/tools). The differentially accumulated metabolites (DAMs) were screened according to the variable importance of the projection (VIP ≥ 1) produced by orthogonal projections to latent structures-discriminant analysis (OPLS-DA) and univariate analysis of variance (ANOVA, *p* < 0.05). 

### 2.7. Enzyme Assay of GiLMT1

A single colony of *E. coli* BL21 (DE3) carrying the *GiLMT1*/*pColdII* vector was incubated in 0.2 mL of Luria–Bertani (LB) medium containing 50 μg/mL ampicillin at 37 °C with agitation at 200 rpm for 16 h. The inoculum was then added into 5 mL medium and cultured until the OD_600_ reached 0.6, followed by the addition of isopropyl-β-ᴅ-thiogalactopyranoside (IPTG) to a final concentration of 0.1 mM. The culture was then incubated at 16 °C with shaking at 140 rpm for 16 h. Cells were collected using centrifugation (4000× *g*, 10 min) and resuspended in 5 mL Tris-HCl (50 mM, pH 7.5). The cells were disrupted using an ultrasonic homogenizer (SCIENTZ-IID) at 50 W for 10 min, and the supernatants were used for enzyme assays.

The crude protein extract (0.1 mL), licodione (200 μM), *S*-adenosyl methionine (SAM, 500 μM, methyl donor), and Tris-HCl buffer (50 mM, pH 7.5) at a total volume of 0.2 mL were incubated at 37 °C for 12 h. After termination of the reaction by adding 0.2 mL methanol, the samples were analyzed using HPLC as described above. The gradient elution approach was set as follows: 0~7 min, 30~45% A; 7~13 min, 45% A; 13~21 min, 45~95% A; 21~24 min, 95% A; 24~25 min, 95~30% A; 25~28 min, 30% A. The standard of licodione was synthesized by WuXi AppTec, Wuxi, China.

### 2.8. Subcellular Localization Analysis of GiSRT2

The subcellular localization of GiSRT2 was studied using transient expression in Arabidopsis protoplasts as previously reported [20].

## 3. Results

### 3.1. Effects of NIC Treatment on Seedling Growth and LCA Accumulation in G. inflata

**Figure 1:**
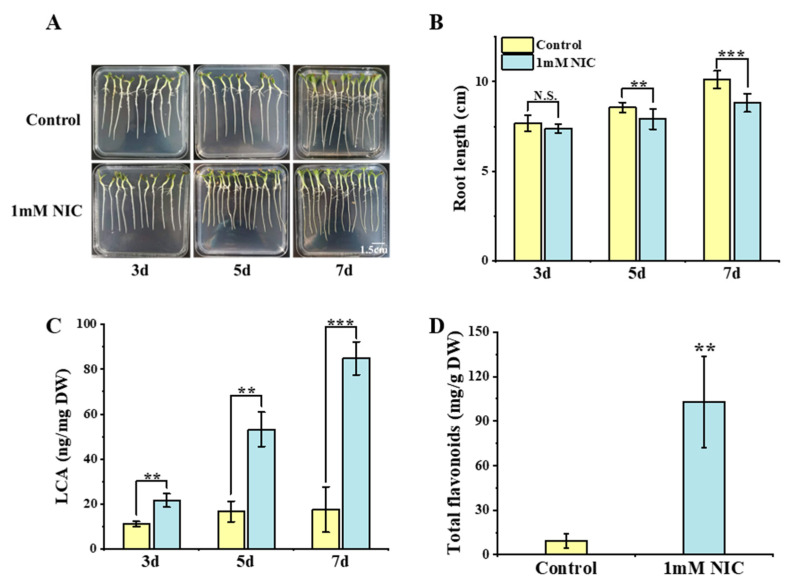
The effects of HDAC inhibitor NIC on seedling growth and flavonoid accumulation in *G. inflata.* (**A**) Phenotype of *G. inflata* seedlings under 1 mM NIC treatment. Upper panel, seedlings of *G. inflata* without treatment (controls); lower panel, (left to right) 7-day-old *G. inflata* seedlings were treated with 1 mM NIC for 3, 5, and 7 days, respectively. (**B**) Root length measurement. (**C**) Measurement of LCA content in roots of seedlings after 1 mM NIC treatment. (**D**) Measurement of total flavonoids in roots of seedlings under 1 mM NIC treatment for 7 days. Values are means ± SD. Student’s *t*-test, n.s. means not significant; ** *p* < 0.01, *** *p* < 0.001, n = 3.

To test the effects of NIC on seedling growth and LCA accumulation, 7-day-old *G. inflata* seedlings were treated with 1 mM NIC on 1/2MS plates. After 3, 5, and 7 days of treatment, NIC significantly inhibited root growth, but resulted in a higher level of LCA than the mock control (Figure 1A–C). Total flavonoids content was also measured 7 days after the treatment. Similar with the result obtained for the characteristic flavonoid LCA, 1 mM NIC promoted the accumulation of total flavonoids, indicating a systemic activation of flavonoid biosynthesis in *G. inflata* roots (Figure 1D). Since NIC is a specific inhibitor of SIR class HDACs, this result suggested that SIR class HDACs are required for normal growth, and they may play negative roles in the regulation of the accumulation of flavonoids, especially LCA.

### 3.2. GiSRT2 Negatively Regulates LCA Accumulation

There are two SIR class HDACs predicted in the *G. inflata* genome named *GiSRT1* and *GiSRT2*. *GiSRT1* was neither detected in 1, 2, or 3-year-old roots nor leaves in the qRT-PCR assay. Whereas, *GiSRT2* was detectable and the expression levels were higher in leaves than in roots (Figure S2A). Since LCA is accumulated in *G. inflata* roots [25] but not detectable in shoots, it suggests that *GiSRT2* is likely the negative regulator of LCA accumulation. We then studied the subcellular localization of GiSRT2-GFP in Arabidopsis protoplast cells. The confocal images showed that the GFP signal of GiSRT2-GFP overlapped with the mCherry signal of NLS-mCherry, indicating the nucleus localization of GiSRT2-GFP (Figure S2B).

**Figure 2:**
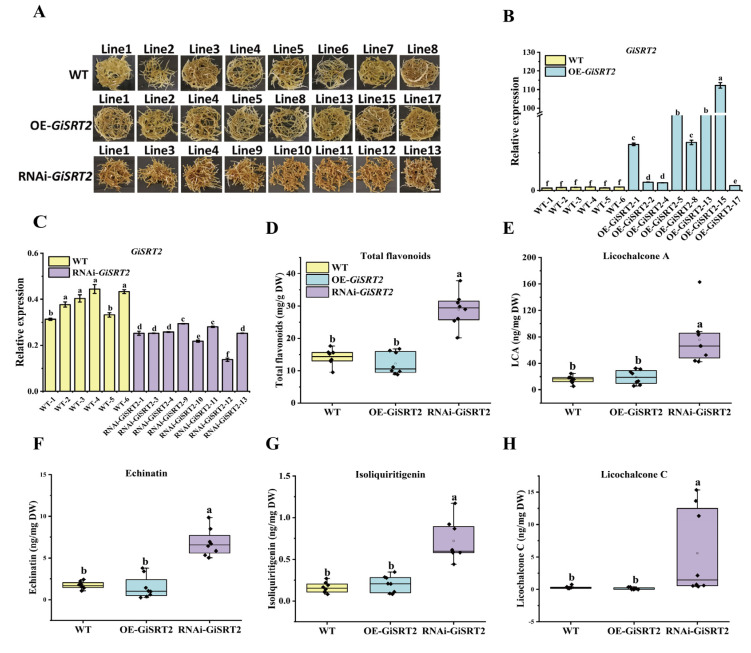
Analysis of flavonoid contents in OE-*GiSRT2* and RNAi-*GiSRT2* transgenic hairy roots. (**A**) The phenotype of the OE-*GiSRT2* and RNAi-*GiSRT2* transgenic hairy roots (scale bars: 1.5 cm). Expression level of *GiSRT2* in the OE-*GiSRT2* (**B**) and RNAi-*GiSRT2* (**C**) lines were detected using qRT-PCR. *GiCOPS3* was used as the internal control. The content of total flavonoids (**D**), LCA (**E**), echinatin (**F**), isoliquiritigenin (**G**), and licochalcone C (**H**) in OE-*GiSRT2* and RNAi-*GiSRT2* transgenic hairy roots were detected using HPLC. The non-transgenic hairy roots are set as the WT. The different lower-case letters indicate significant differences at a *p* value of 0.05 for the relative expression level and the contents of flavonoids among samples. Student’s *t*-test, n ≥ 3.

To verify the function of *GiSRT2* in the regulation of LCA accumulation, we generated OE-*GiSRT2* and RNAi-*GiSRT2* transgenic hairy roots of *G. inflata*. The transgenic hairy root lines were confirmed using PCR and qRT-PCR assays (Figure S3 and Figure 2B,C). Since the growth of hairy roots or accumulation of specialized metabolites among WT (generated by infection with MSU440), OE-CK (generated by infection with MSU440 carrying *pSuper-GFP* empty vector), and RNAi-CK (generated by infection with MSU440 carrying *pRNAiGG* empty vector) (Figure S1) were similar, we used WT transgenic hairy roots as the control in this part. The *RNAi*-*GiSRT2* hairy roots exhibited a darker color compared to the control and OE-*GiSRT2* lines, consistent with the higher level of total flavonoids in RNAi-*GiSRT2* lines (Figure 2A,D). Furthermore, the RNAi-*GiSRT2* transgenic hairy roots accumulated a higher level of LCA when compared with the WT control lines and OE-*GiSRT2* lines. Based on the reported data, we proposed a hypothetical LCA biosynthesis pathway (Figure S4) [9,10,25]. The contents of specialized flavonoids in this pathway including LCA precursor echinatin, its isomer licochalcone C (LCC), and other reported bioactive flavonoid isoliquiritigenin in licorice roots were measured. HPLC results showed that the contents of echinatin and isoliquiritigenin, as well as LCC, were much higher in RNAi-*GiSRT2* lines than those in the control and OE-*GiSRT2* lines (Figure 2D–F). These results demonstrated that *GiSRT2* negatively regulated the accumulation of LCA and total flavonoids in *G. inflata* hairy roots.

### 3.3. Transcriptome Sequencing (RNA-Seq) Analysis of RNAi-GiSRT2 Lines

To unveil the mechanism underlying the enhanced LCA biosynthesis in RNAi-*GiSRT2* transgenic hairy roots, the transcriptome sequencing was carried out. Principal component analysis (PCA) of the samples showed that the CK (RNAi empty vector-induced hairy roots) and RNAi-*GiSRT2* sample types were grouped well and separated clearly, indicating the high quality of the generated transcriptome data (Figure S5A). The significant DEGs were selected by setting |log_2_FC| ≥ 1 and *p*-adjust ≤ 0.001 as the thresholds. As shown in the volcano plot, a total of 4930 significant DEGs were identified in the RNAi-*GiSRT2*_VS_CK pair (Figure S5B). Hierarchical cluster analysis showed that the expression patterns of most DEGs in CK and RNAi-*GiSRT2* were completely opposite, and indicated good repeatability between biological replicates (Figure S5C).

**Figure 3:**
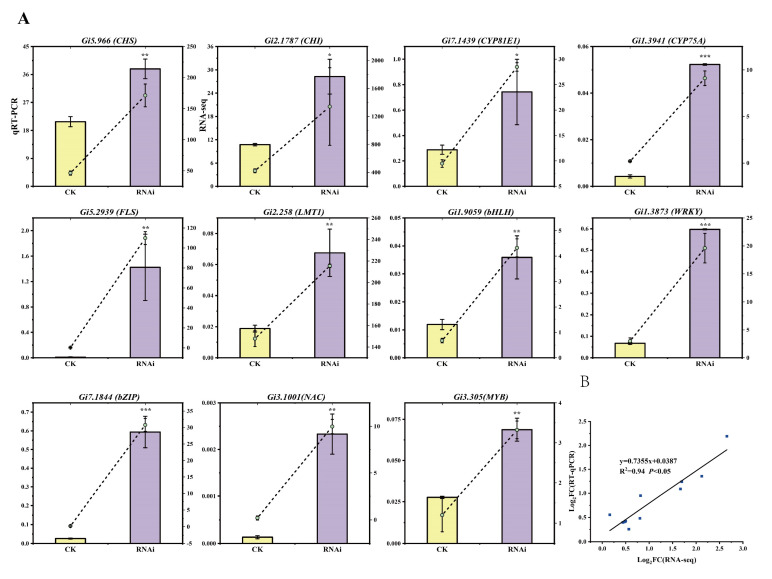
qRT-PCR verification of expression patterns of 11 DEGs related to flavonoid biosynthesis. (**A**) The results of qRT-PCR with 11 genes. The *CHS*, *CHI*, *CYPs*, *FLS*, and *LMT* are structural genes in flavonoid biosynthetic pathways. CHS, chalcone synthase; CHI, chalcone isomerase; CYP, cytochrome P450; FLS, flavonol synthase; LMT, licodione 2′-*O*-methyltransferase. bHLH, WRKY, bZIP, NAC, and MYB are transcription factors. *GiCOPS3* was used as the internal control. The relative expression levels of genes in transcriptome data and qRT-PCR are shown as dashed lines and bars, respectively. Values are shown as means ± SD. (Student’s *t*-test, * *p* < 0.05, ** *p* < 0.01, *** *p* < 0.001, n = 3). (**B**) Correlation analysis between RNA-Seq and qRT-PCR results (R^2^ = 0.94, *p* < 0.05). RNAi-EV induced hairy roots are used as the control.

A total of five differentially expressed TFs and six differentially expressed structural genes were selected for qRT-PCR to verify the reliability of RNA sequencing. As shown in Figure 3A, the expression levels of the selected genes displayed high consistency with the RNA-seq data, and the RNA sequencing data correlated significantly with the qRT-PCR results (R^2^ = 0.94, *p* < 0.05; Figure 3B).

To elucidate the function of the DEGs, the GO and KEGG enrichment analysis were carried out. As shown in Figure S6A, the annotated DEGs were classified into 14 subclasses under molecular function, 15 subclasses under biological process, and 2 subclasses under cellular component. Among them, ‘cellular process’, ‘metabolic process’, and ‘response to stimulus’ were the most significantly enriched BPs. The majority of GO terms in cellular component enrichments occurred in ‘cellular anatomical entity’ and molecular function enrichments primarily occurred in ‘binding’ and ‘catalytic activity’. KEGG annotation results showed that DEGs were assigned to 130 pathways, among these pathways, the enriched pathways were significantly concentrated in ‘phenylpropanoid biosynthesis’, ‘isoflavonoid biosynthesis’, and ‘biosynthesis of secondary metabolites’ (Figure S6B).

Our RNA-seq analysis revealed that the DEGs contained 252 transcription factor-encoding genes, of which 171 showed up-regulation and 81 down-regulation (Figure S7A). They could be divided into 48 different common families. These families contain a significant number of TFs, particularly the AP2/ERF (12.7%), bHLH (9.52%), and MYB-related (8.33%) families (Figure S7B); the expression levels of identified TF family genes are shown by heatmap. The expression of some genes of WRKY, MYB, and AP2/ERF TFs was higher in RNAi-*GiSRT2* lines than in the controls (Figure S7C), indicating that these genes might play a regulatory role in the flavonoid metabolism.

### 3.4. Metabolome Analysis of RNAi-GiSRT2 Lines

To reveal the regulatory role of *GiSRT2* in the whole metabolism, a widely targeted metabolomics assay was carried out using CK and RNAi-*GiSRT2* hairy roots. We used an ultra-performance liquid chromatography-tandem mass spectrometry (UPLC-MS) method to identify changes in metabolite levels. PCA indicated that the metabolites of different genotypes were significantly different (Figure S8A). A total of 1360 metabolites were detected with this approach, including 12 different types of substances, among these metabolites, with 172 phenolic acids, 351 flavonoid metabolites, 171 organic acids, 141 lipids, and 135 terpenoids (Figure S8B). Cluster analysis was also performed with twelve samples being clearly divided into four groups, indicating significant differences in metabolites among four experiment groups (Figure S8C).

Based on thresholds (|log_2_FC| ≥ 1 and VIP ≥ 1), we obtained 376 DAMs between CK and RNAi-*GiSRT2* (Figure S9A). Among them, 194 metabolites were up-regulated and 182 metabolites down-regulated in RNAi-*GiSRT2* hairy root lines. Notably, isoflavonoids and flavonoids were up-regulated in RNAi-*GiSRT2*. The overlap analysis of the DAMs showed that 214 DAMs were overlapped between the 4 groups (Figure S9B). KEGG enrichment analysis revealed that “alpha-Linolenic acid metabolism” (ko00592), “Linoleic acid metabolism” (ko00591), and “Isoflavonoid biosynthesis” (ko00943) were significant (Figure S9C).

### 3.5. Combined Analysis of Transcriptome and Metabolome

To investigate the relationship of DEGs and DAMs annotated with KEGG pathways, we first calculated the Pearson correlation coefficients of DEGs and DAMs. Pearson’s correlation coefficient ≥ 0.8 was considered to be significantly correlated. Then, the significantly correlated DEGs and DAMs were mapped onto the KEGG pathway database to gather information about their shared pathways to better show their interaction. The KEGG pathway enrichment analysis showed that the DEGs and DRMs were commonly enriched in 18 KEGG pathways, such as the flavonoid, phenylpropanoid, linoleic acid metabolism, diterpenoid biosynthesis, riboflavin biosynthesis, and isoflavonoid biosynthesis (Figure S10A). Among the 18 KEGG pathways, the isoflavonoid biosynthesis and phenylpropanoid biosynthesis pathways were significantly enriched. To reveal the correlation between the DEGs and DRMs involved in these two pathways, the networks between the DEGs and DRMs were constructed using the screening criteria of the absolute values of PCC ≥ 0.99 and *p* < 0.01 (Figure S10B,C). The significance of flavonoid biosynthesis in RNAi-*GiSRT2* hairy root lines was highlighted using transcriptome analysis, so this metabolic pathway was focused on in later analyses.

### 3.6. Analysis of UDEGs Involved in Flavonoid Biosynthetic Pathways in RNAi-GiSRT2 Hairy Roots

**Figure 4:**
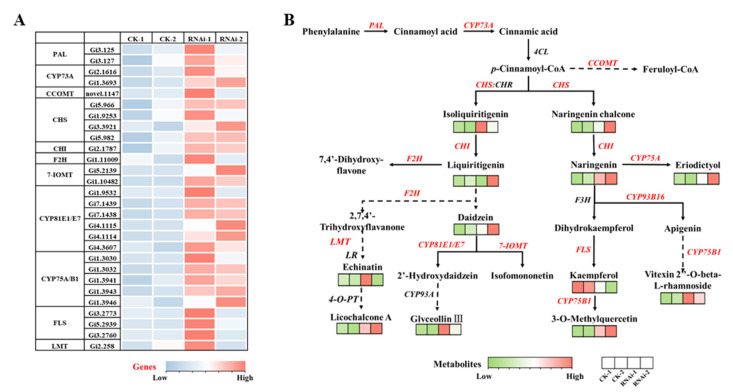
The DEGs and DAMs in flavonoid biosynthetic pathways. (**A**) Heat map of gene expression levels of key enzymes in flavonoid biosynthetic pathways. PAL, phenylalanine ammonia lyase; CYP73A, *trans*-cinnamate 4-monooxygenase; CCOMT, caffeoyl CoA *O*-methyltransferase; CHS, chalcone synthase; CHR, chalcone reductase; F2H, flavanone 2-hydroxylase; CYP75A, flavonoid 3′,5′-hydroxylase; FLS, flavonol synthase; CHI, chalcone isomerase; 7-IOMT, isoflavone 7-*O*-methyltransferase; CYP75B1, flavonoid 3′-monooxygenase; LMT, licodione 2′-*O*-methyltransferase. (**B**) Heat map of metabolites in flavonoid biosynthetic pathways. RNAi-EV induced hairy roots are used as the control.

It has been widely reported that a series of structural genes, including phenylalanine ammonia lyase genes (*PAL*s), cytochrome P450 genes (*CYP*s), chalcone synthase genes (*CHS*s), chalcone isomerase genes (*CHI*s), flavanone 2-hydroxylase (*F2H*s), *O*-methyltransferase genes (*OMT*s), and flavonol synthase genes (*FLS*s), co-regulated flavonoid biosynthesis in plants. In the present study, the biosynthetic pathways of flavonoid and up-regulated DEGs (UDEGs) in RNAi-*GiSRT2* hairy roots are summarized (Figure 4). In phenylpropanoid and flavonoid biosynthetic pathways, two *PAL*s, fourteen *CYP*s, four *OMT*s, four *GiCHS*s, one *GiCHI*, and three *GiFLS*s were up-regulated. These results were well consistent with the enhanced accumulation of LCA and other flavonoids in RNAi-*GiSRT2* lines. This suggested that the above genes are potential key ones regulating flavonoids biosynthesis in RNAi-*GiSRT2* hairy roots.

### 3.7. GiLMT1 Is Involved in the Biosynthesis of LCA in G. inflata

To identify the genes involved in the biosynthesis of LCA, DEGs in the proposed biosynthetic pathway (Figure S4) were investigated. Up-regulated *PAL*s, *CYP*s, *CHS*s, *CHI*, *F2H*, and *LMT* may be involved in the biosynthesis of precursors (Figure 4A). Here we selected the LMT that was predicted to catalyze the next steps in the pathway, which is more likely to be involved in LCA biosynthesis. We cloned the coding sequence of *Gi2.258* and named it *GiLMT1.* It encodes a protein which shares 83.60% identity with MsLMT (Figure S11), the first cloned LMT from *Medicago sativa* L. [26]. The expression level of *GiLMT1* was higher in roots than in leaves, contrary to *GiSRT2*, suggesting the opposite effects of *GiLMT1* and *GiSRT2* in LCA accumulation (Figure S12).

**Figure 5:**
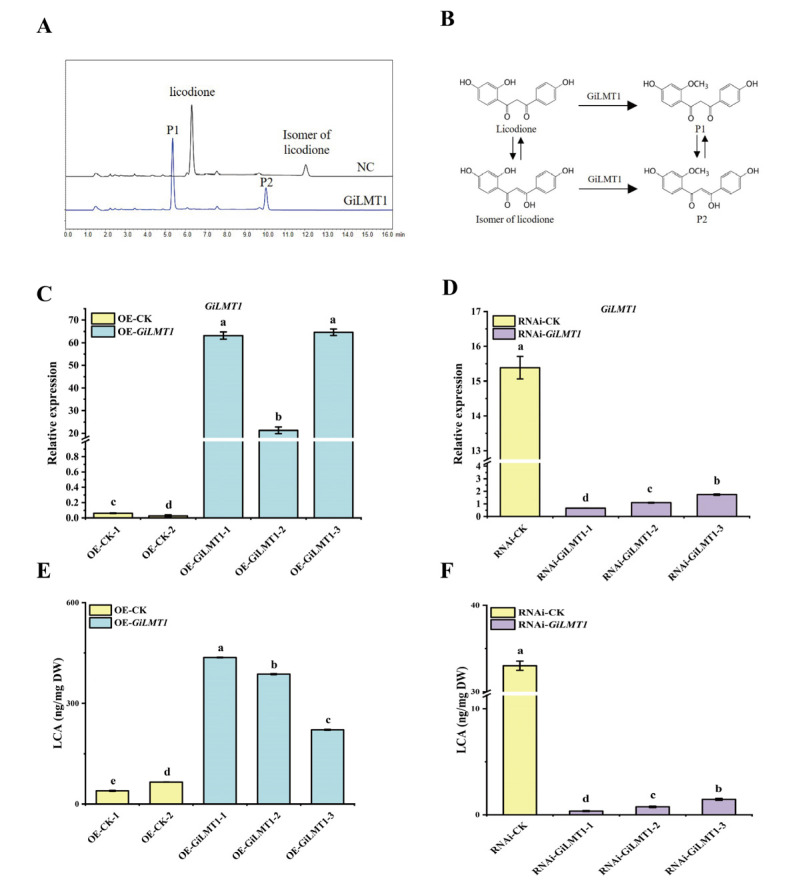
GiLMT1 was required for LCA biosynthesis. (**A**) HPLC profiles of enzyme reaction products formed by the recombinant GiLMT1 protein with licodione and its isomer as substrates. NC, negative control, reaction catalyzed by crude protein extract of *E. coli* carrying empty vector. (**B**) The reactions catalyzed by GiLMT1 that converted licodione and its isomer to 2′-*O*-methyllicodione (P1) and P2, respectively. (**C**,**D**) qRT-PCR verification of expression level of *GiLMT1* in OE-*GiLMT1* (**C**) and RNAi-*GiLMT1* (**D**) lines. *GiCOPS3* was used as the internal control. (**E**,**F**) Measurement of LCA contents in OE-*GiLMT1* (**E**) and RNAi-*GiLMT1* (**F**) transgenic hairy roots. The different lower-case letters indicate a significant difference at the 0.05 level for the relative expression level and LCA content among samples. Student’s *t*-test, n = 3.

To verify the function of GiLMT1, we carried out in vitro enzyme assays. The crude enzyme of GiLMT1 obtained by heterologous expression in *E. coli* was incubated with licodione and *S*-adenosyl methionine (SAM), the methyl donor. GiLMT1 could transfer a methyl group to licodione and produce 2′-*O*-methyllicodione (**P1**) (Figure 5A,B). Moreover, licodione is unstable and interconvertible with its isomer. GiLMT1 was also capable of catalyzing the licodione isomer, yielding the methylated product **P2** (Figure 5A,B). The substrates and products were all confirmed using mass spectrometry (Figure S13). These results indicated that GiLMT1 could catalyze the methylation of licodione in vitro.

To test the function of GiLMT1 in vivo, OE-*GiLMT1* and RNAi-*GiLMT1* transgenic hairy roots were generated and verified using qRT-PCR (Figure 5C,D). Compared with hairy roots carrying *pSuper-GFP* empty vector, the OE-*GiLMT1* lines displayed increased LCA contents (Figure 5E), which is similar to the case observed in RNAi-*GiSRT2* lines. In contrast, the LCA contents in RNAi-*GiLMT1* lines were significantly lower than those in the control lines (Figure 5F). These results indicated that GiLMT1 is involved in the biosynthesis of LCA in *G. inflata*. However, more research is needed to determine whether *GiLMT1* is a direct target of *GiSRT2*.

## 4. Discussion

LCA is a characteristic flavonoid in *G. inflata*, a medicinal licorice species. Despite its high economic value, the biosynthetic pathway and the molecular regulation mechanism are not clear. Identification of enzymes involved in LCA biosynthesis will help to reconstruct the pathway in different organisms for the production of LCA. Furthermore, discovering factors that affect LCA accumulation would help to improve LCA content in farmed licorice.

### 4.1. The Role of GiSRT2 in Balancing Growth and Resistance of G. inflata

A previous study found that when ginseng adventitious roots were treated with HDAC inhibitors SAHA or NaB, the MeJA-induced H3ac level was significantly increased, and the inhibition of HDAC activity improved MeJA-induced transcriptional activation of ginsenoside biosynthesis pathways (Lu and Hyun, 2021). Here we found that NIC treatments significantly increased the content of total flavonoids and LCA in *G. inflata* seedlings (Figure 1C,D), which is consistent with the observation in the OE/RNAi-GiSRT2 transgenic hairy roots (Figure 2D,E), suggesting that GiSRT2 is a negative regulator of flavonoid biosynthesis. Inhibition of GiSRT2 activity by NIC treatment or RNAi released the repression on flavonoid biosynthesis. Accumulation of flavonoids has been reported to be important protection against different environmental stresses such as UV, drought, etc. [27]. In white clover, different flavonoids induced in cells undergoing nodule organogenesis regulated local auxin levels either by promoting or repressing auxin breakdown and thus regulated root growth [28]. Since NIC treatment inhibited root growth but promoted flavonoid accumulation, our results indicated that GiSRT2 is an important regulator to balance plant growth and stress responses/accumulation of flavonoids. However, despite its inhibitory effect on plant growth, NIC is still a potential ‘fertilizer’ that could be used in the licorice field before root harvest, when certain biomass has been accumulated and a short NIC treatment may activate the LCA biosynthesis resulting in a better quality of licorice.

### 4.2. Determination of Gene Expression and Metabolic Changes in RNAi-GiSRT2 Hairy Roots through Transcriptomic and Metabolomic Techniques

Since the molecular mechanism of HDAC regulating flavonoid biosynthesis in plants is rarely reported, we carried out integrated transcriptomic and metabolomic analysis with RNAi-*GiSRT2* transgenic hairy roots. Structural genes affecting specialized metabolism of *G. inflata* and metabolic pathways with significant enrichment were analyzed. A total of 4930 DEGs were identified in RNAi-*GiSRT2.* PAL, CYP73A, and CCOMT are associated with lignin biosynthesis. Twenty-three DEGs were annotated to the flavonoid biosynthesis pathway, including *CHS*s, *CHI*, *F2H*, *FLS*, and *LMT*. Consistently, the contents of 11 DAMs were mapped to flavonoid biosynthesis and accumulated more in RNAi-*GiSRT2* hairy roots. Furthermore, the KEGG pathway enrichment analysis showed that the DEGs and DRMs were commonly enriched in 18 KEGG pathways involved in the phenylpropanoid, linoleic acid metabolism, diterpenoid biosynthesis, and riboflavin biosynthesis (Figure S10A). These results not only lay the foundation for the excavation of functional genes related to the biosynthesis of LCA in *G. inflata*, but also provide an overall picture of sirtuins regulating plant specialized metabolism. Further study on sirtuins regulating the biosynthesis of different specialized metabolites could be carried out based on these omics data.

### 4.3. GiLMT1 Is Required for LCA Biosynthesis

In this work, *GiLMT1* was found up-regulated in RNAi-*GiSRT2* lines. The in vitro enzyme assays showed that GiLMT1 could add a methyl group to licodione, which is an intermediate step in LCA biosynthesis. Consistently, the transgenic hairy roots overexpressing GiLMT1 produced more LCA while the LCA contents were greatly reduced in RNAi lines. Both in vitro and in vivo data demonstrated the key role of GiLMT1 in the LCA biosynthesis process. Our work provides a key point that could be used in both molecular breeding of licorice with high LCA content, and in synthetic biology to produce LCA in different organisms. It is true that we cannot rule out the possibilities that other LMTs may be involved in LCA biosynthesis and more studies are needed to figure precisely out how GiSRT2 regulates the expression of *GiLMT1*.

## 5. Conclusions

In this study, we proved that an SIR class HDAC-specific inhibitor NIC could increase the content of flavonoids in *G. inflata* roots. We further cloned and characterized a NIC-targeted HDAC gene, *GiSRT2*, which is predominantly located in the nucleus and negatively regulates the accumulation of flavonoids in hairy root transgenic lines. Combined analysis of metabolome and transcriptome with RNAi-*GiSRT2* transgenic hairy roots revealed the potential mechanism underlying this process. The expression level of several structural genes of flavonoid biosynthesis pathways in RNAi-*GiSRT2* hairy roots was up-regulated, among which *GiLMT1* was proven to be required for the biosynthesis of LCA in *G. inflata*. This study provides new insights into the role of GiSRT2 in the regulation of the biosynthesis of specialized metabolites in *G. inflata*. It also provides evidence for the possibility of a novel metabolic engineering strategy to promote important specialized metabolite production in medicinal plants.

## Data Availability

The raw sequence data reported in this paper have been deposited in the Genome Sequence Archive (Genomics, Proteomics & Bioinformatics 2021) in National Genomics Data Center (Nucleic Acids Res 2022), China National Center for Bioinformation/Beijing Institute of Genomics, Chinese Academy of Sciences (GSA: CRA011103) that are publicly accessible at https://ngdc.cncb.ac.cn/gsa.

## Supplementary Materials

The following supporting information can be downloaded at: https://www.mdpi.com/article/10.3390/cells12111501/s1, Figure S1: Analysis of flavonoid contents in non-transgenic hairy roots (WT), OE empty vector induced hairy roots (OE-CK), RNAi empty vector induced hairy roots (RNAi-CK); Figure S2: Expression pattern and subcellular localization of GiSRT2; Figure S3: Identification of OE-GiSRT2 and RNAi-GiSRT2 transgenic hairy roots; Figure S4: Proposed biosynthetic pathway of LCA in G. inflata; Figure S5: Gene expression analysis of RNA-seq samples; Figure S6: Gene functional enrichment analysis of DEGs; Figure S7: Analysis of differentially expressed transcription factors; Figure S8: Analysis of the metabolites detected by the metabolome; Figure S9: Overall analysis of the metabolomics data of RNAi-GiSRT2; Figure S10: Correlation analysis between DAMs and DEGs; Figure S11: Amino acid sequence alignment of GiLMT1 and MsLMT; Figure S12: Tissues expression pattern of GiLMT1 in G. inflata; Figure S13: Identification of licodione, its isomer and their methylated products by mass spectrum; Table S1: Primers used in this study.

## Abbreviations

7-IOMT	isoflavone 7-*O*-methyltransferase
CCOMT	caffeoyl CoA *O*-methyltransferase
CDS	coding sequence
CHI	chalcone isomerase
CHR	chalcone reductase
CHS	chalcone synthase
CYP73A	*trans*-cinnamate 4-monooxygenase
CYP75A	flavonoid 3′,5′-hydroxylase
CYP75B1	flavonoid 3′-monooxygenase
DAM	differentially accumulated metabolite
DEG	differentially expressed gene
F2H	flavanone 2-hydroxylase
FLS	flavonol synthase
HDAC	histone deacetylase
HPLC	high performance liquid chromatography
LCA	licochalcone A
LCC	licochalcone C
LMT	licodione 2′-*O*-methyltransferase
NIC	nicotinamide
PAL	phenylalanine ammonia lyase
SAM	*S*-adenosyl methionine
