# Supplementary material for: Histone Deacetylase GiSRT2 Negatively Regulates Flavonoid Biosynthesis in Glycyrrhiza inflata
Source: Cells. 2023 May 29;12(11):1501. doi: 10.3390/cells12111501 (PMC10252568; doi:10.3390/cells12111501)
Supplement: Supplementary file 1 [file cells-12-01501-s001.zip › cells-2309778-supplementary.pdf]

Supplementary Table S1. Primers used in this study

Gene	Primer sequence (5'-3')
GiSRT2 (pSuper1300)	F:ACGCGTCGACATGAAGGATATTGTTGTTAGTCCA<br>R:GGGGTACCCACAGAAGGTATGCTGATGGAT
GiSRT2 (pCambia1300)	F:GATTAACAGGGATCCCCCATGAAGGATATTGTTGTT<br>R:GAGACTAGTGGTACCCCCCACAGAAGGTATGCTGAT
GiLMT1 (pSuper1300)	F:AACTGCAGATGGGGGATTCATATTCCAC<br>R:GGACTAGTTTTATAAAATTCCATCACTCC
GiLMT1 (pRNAiGG)	F:ACCAGGTCTCAGGAGGATTTTGTCCATTTCCACCT<br>R:ACCAGGTCTCATCGTTCCATTCTCACTTGTTCCAC
GiSRT2 (pRNAiGG)	F:ACCAGGTCTCAGGAGCCCGACTATAGAAGCCCCAA<br>R:ACCAGGTCTCATCGTCCACTTTGGATTAAGGTCCTTC
GiLMT1 (pColdII)	F:AGCGAGCTCATGGGGGATTCATATTCCAC<br>R:ACGCGTCGACTTATAAAATTCCATCACTCC
GiCOPS3	F: GGAAGCGCCAATACGAGG<br>R: ACAACAAGCACAGCAGAAGAAA
rolB	F: CTTATGACAAACTCATAGATAAAAGGTT<br>R: TCGTAACTATCCAACTCACATCAC
Gi5.966 (CHS)	F:ACTTTAAGATCACAAACAGTGA<br>R:TCCAAAGAAGGTGCCATATAA
Gi2.1787 (CHI)	F:CCCTGATGGAATATTAGGGC<br>R:AAACAGCGTTTCAAATCAGG
Gi7.1439 (CYP81E1)	F:TACCCCATGAGTCCTCTAAG<br>R:GTTAGCATCCCTCTGTAGTG
Gi1.3941 (CYP75A)	F:GTTCCATGCATTTTTGACCA<br>R:AGAAGTCCTTTGATCTCGTG
Gi5.2939 (FLS)	F:GTAGCTCATGAGTCCAAACA<br>R:TGGGTCGCTGAAATCAATTA
Gi2.258 (LMT)	F:TGCCACTCGCAGCACACAG<br>R:CCAAGTATCCCCTGCTTCCAT
Gi1.9059 (bHLH)	F:GTGTTGGGGGATGCTATAAA<br>R:ATATCATCGGAGCTGTTTCC
Gi1.3873 (WRKY)	F:TTCCAAGTAACCCAATAGCC<br>R:CAGCTTCTTCCTTTGGTACT
Gi7.1844 (bZIP)	F:GCGTGCTAAAAGGATATTGG<br>R:CTGCTTTTAGTGATTGCACC
Gi3.1001 (NAC)	F:GAGGGCATGTTTAATCCACT<br>R:CATGTCTTAGCTTCCCATCA
Gi3.505 (MYB)	F:CTAGAGAGCGAGAATTGGTC<br>R:ACTTTCTTTCTCACCGTCAA

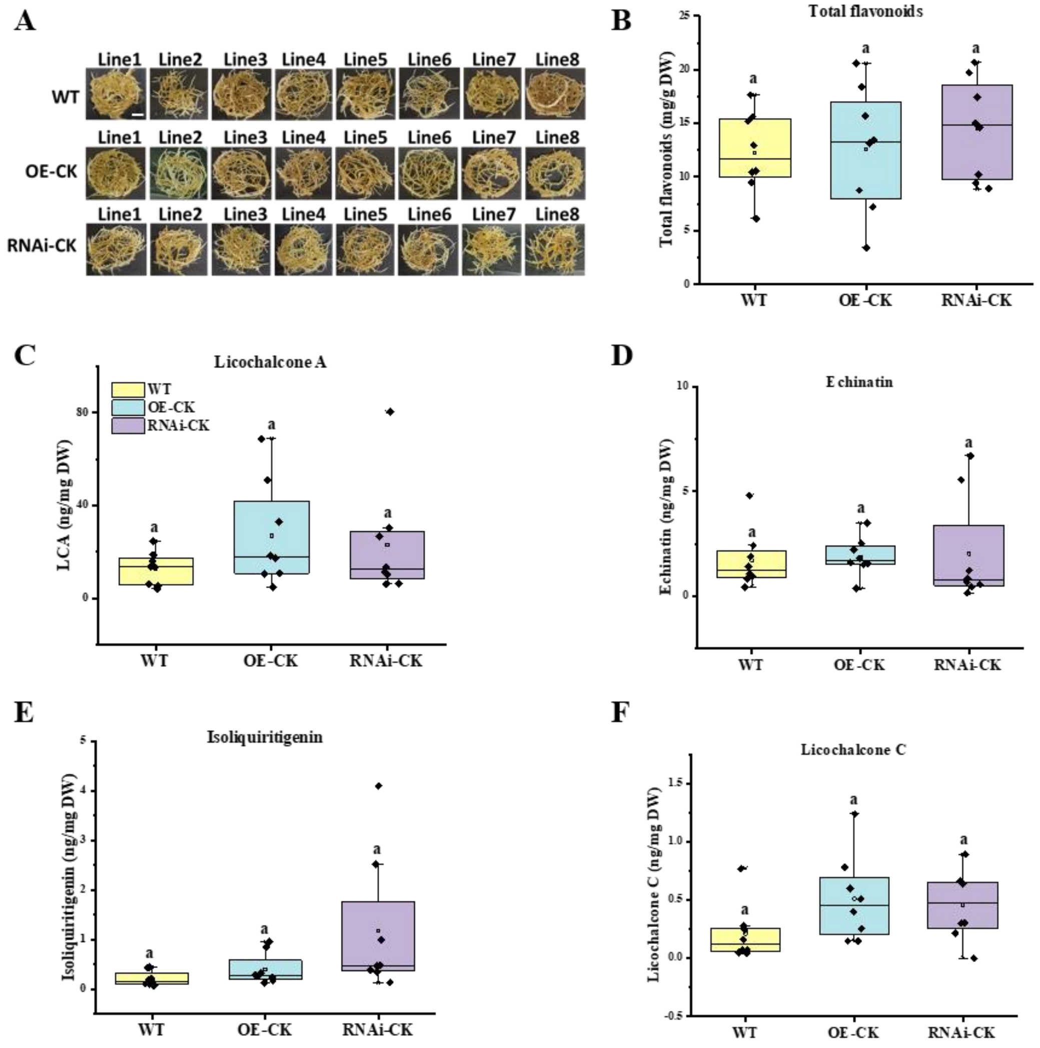

Figure S1. Analysis of flavonoid contents in non-transgenic hairy roots (WT), OE empty vector induced hairy roots (OE-CK), RNAi empty vector induced hairy roots (RNAi-CK). (A) The phenotype of the WT, OE-CK and RNAi-CK hairy roots (scale bars: 1.5 cm). The contents of total flavonoids (B), LCA (C), echinatin (D), isoliquiritigenin (E) and licochalcone C (F) in WT, OE-CK and RNAi-CK hairy roots were detected by HPLC. The lower-case "a" letter indicated no significant difference in the content of flavonoids among samples at the *P* value level of 0.05. Student's *t*-test, * $P < 0.05$, n = 8.

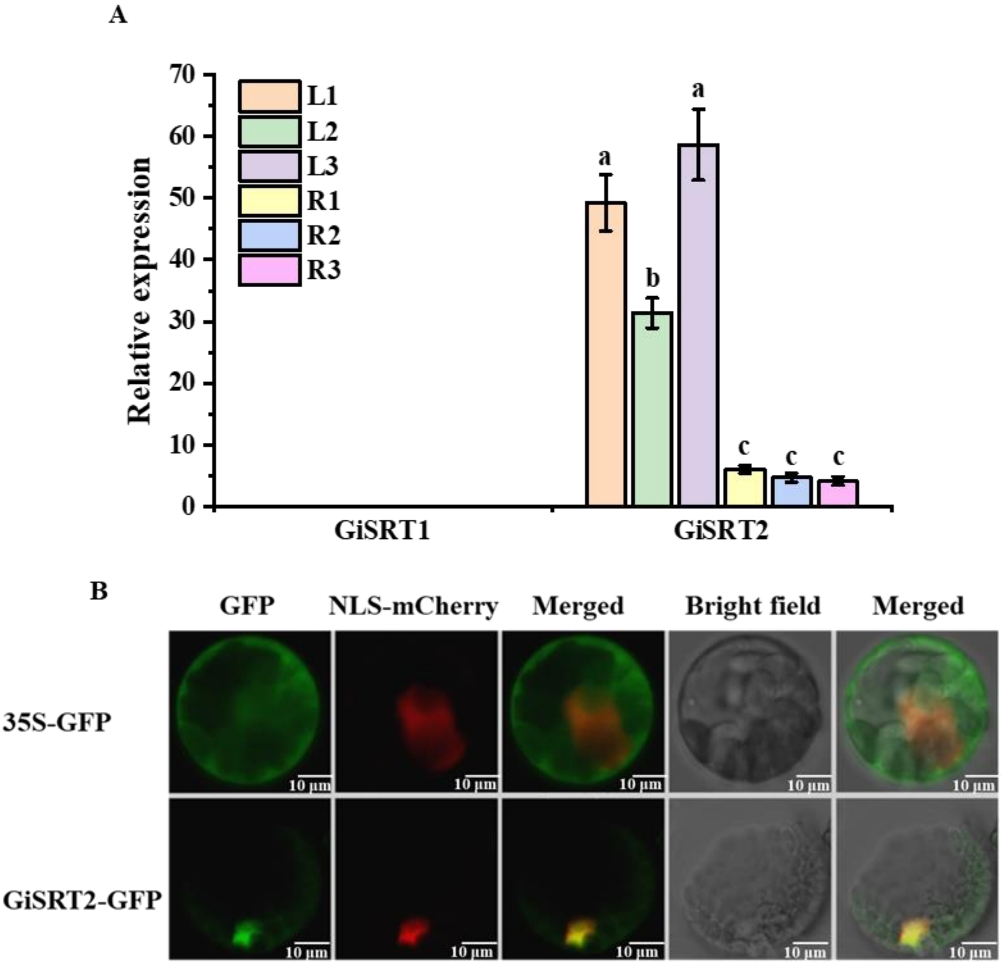

Figure S2. Expression pattern and subcellular localization of *GiSRT2*. (A) The expression levels of the *GiSRT1* and *GiSRT2* from leaves (L1~L3) and roots (R1~R3) of *G. inflata* at different developmental stages (one/two/three-year(s)-old, respectively) were detected by qRT-PCR. The different lower-case letter indicated significant difference at 0.05 level for the relative expression level among samples. Student's *t*-test, * $P < 0.05$, n = 3. (B) Subcellular localization of *35S:GiSRT2::GFP* and *35S:GFP* in *Arabidopsis* leaf protoplasts. Scale bars: 10 μm.

A

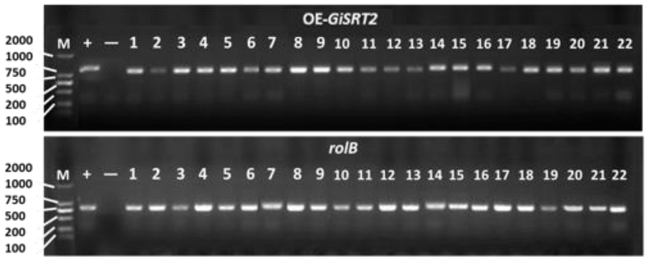

B

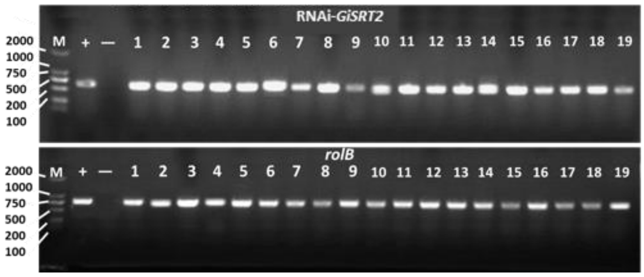

Figure S3. Identification of OE-*GiSRT2* and RNAi-*GiSRT2* transgenic hairy roots. Identification of OE-*GiSRT2* (A) and RNAi-*GiSRT2* (B) hairy roots by PCR. M, DNA marker. The plasmid DNA from MSU440 strain carrying *pSuper*-*GiSRT2* vector was used as positive control and labelled as "+". The seedling without transgene was used as negative control and labelled as "-". The expected sizes of PCR products of OE-*GiSRT2*, RNAi-*GiSRT2* and *rolB* were 1095 bp, 621 bp and 738 bp, respectively.

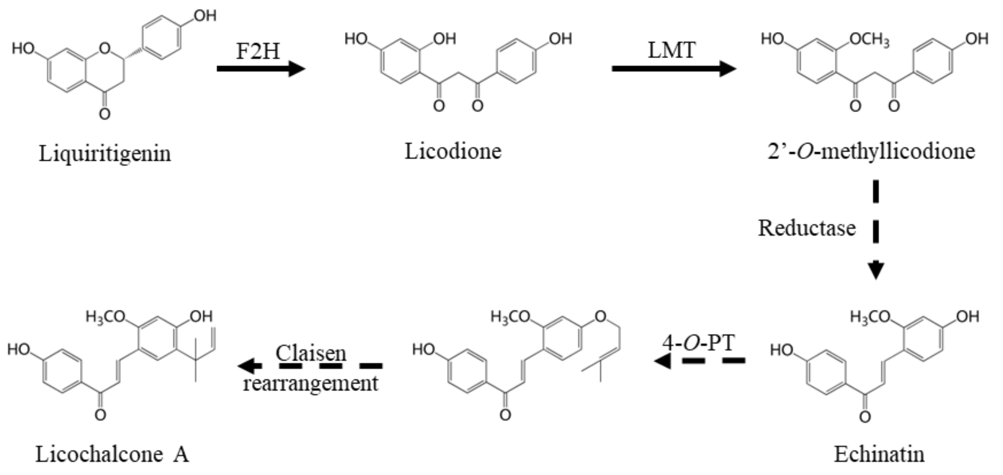

Figure S4. Proposed biosynthetic pathway of LCA in *G. inflata*. Liquiritigenin, a common precursor of chalcones in licorice, was proposed to be converted to LCA in four or five steps. Flavanone 2-hydroxylase (F2H) and licodione 2′-*O*-methyltransferase (LMT) were reported to be involved in the biosynthesis of echinatin in *G. echinata* (Otani et al., 1994; Ayabe et al., 1980). Following LMT, a reductase was proposed to reduce 2′-*O*-methyllicodione to echinatin. The close structural similarity of echinatin and LCA suggests that echinatin is the direct precursor of LCA. It is very likely that the final steps of LCA biosynthesis are catalyzed by a 4-*O*-prenyltransferase (4-*O*-PT) which adds a prenyl group to the 4-OH of echinatin and followed by claisen rearrangement to generate LCA.

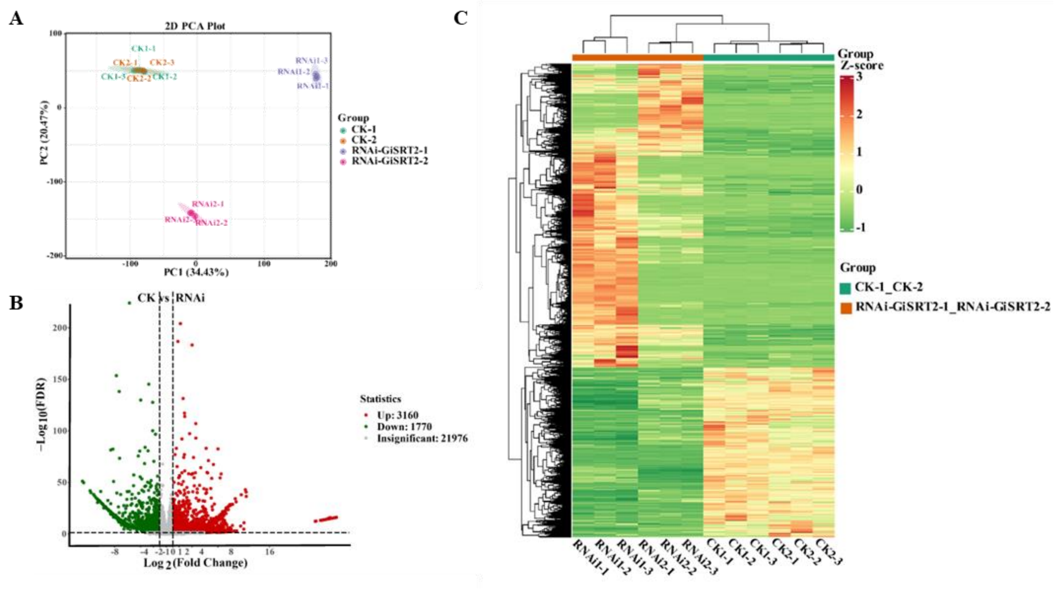

Figure S5. Gene expression analysis of RNA-seq samples. (A) Principal component analysis (PCA) based on FPKM data. (B) Volcano plot of DEGs in CK and RNAi-*GiSRT2*. Each point represents a

DEG. In the plots, 2-based log gene expression level ratios were plotted against the 10-based log false discovery rate (FDR). Genes with FDR < 0.05 and 2-based log fold change above 1 were considered to be up-regulated genes shown in red. Genes with FDR < 0.05 and 2-based log fold change below -1 were considered to be down-regulated genes shown in green. The genes in gray did not show differential expression. (C) Clustering analysis of DEGs between the CK and RNAi-*GiSRT2* samples. The color scale from green to red in the heatmap represents the normalized FPKM value using the Row Z score. RNAi-EV induced hairy roots were set as CK.

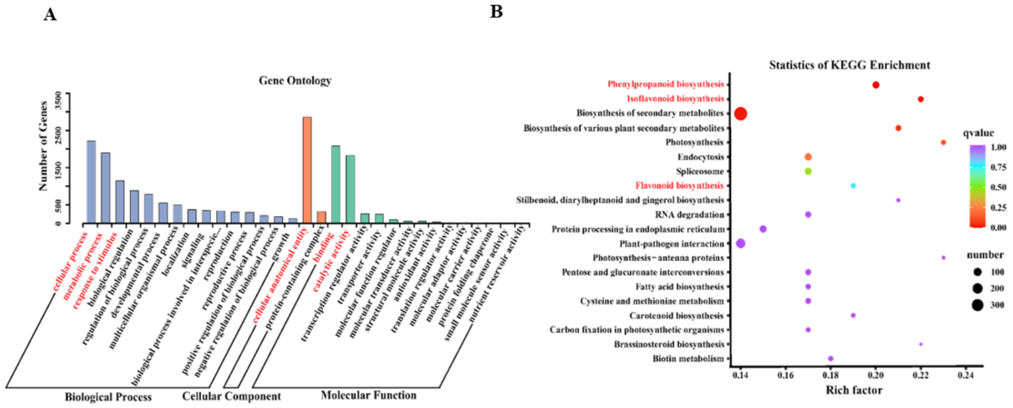

Figure S6. Gene functional enrichment analysis of DEGs. (A) Enrichment of the top 20 GO pathways of all upregulated genes (3160) according to the *p*-value (< 0.05). (B) Top 20 KEGG pathways enriched in the annotated DEGs. Rich ratio represents the ratio of differential expression for the top 20 DEGs. Rich factor is the ratio of the proportion of DEGs annotated to a pathway among all DEGs to the proportion of genes annotated to that pathway among all genes; the red represents significant enrichment.

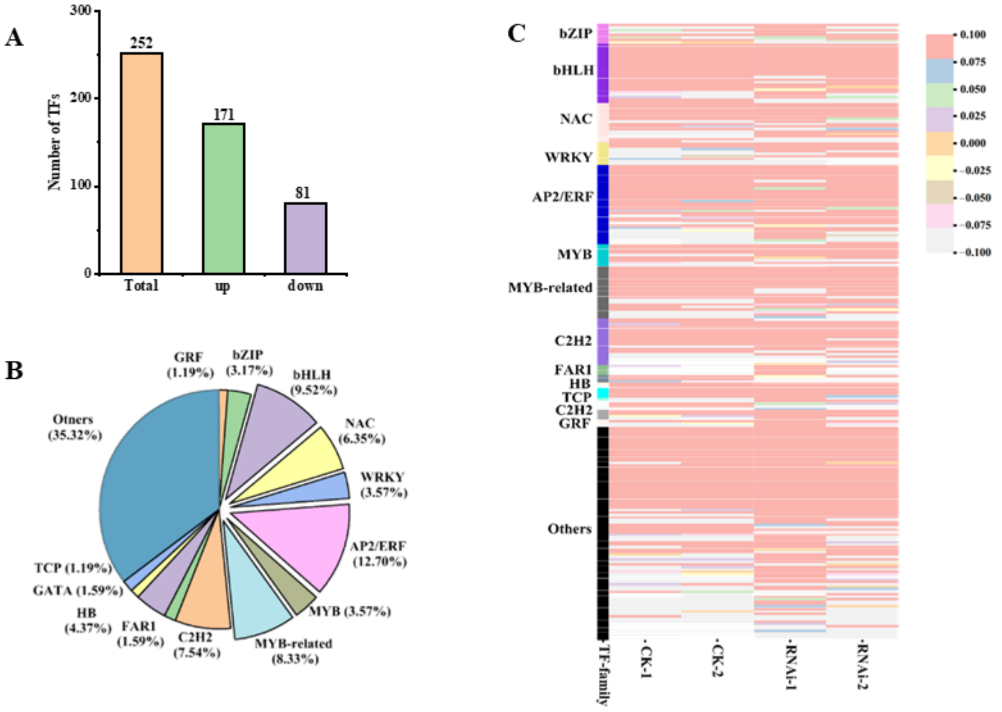

Figure S7. Analysis of differentially expressed transcription factors. (A and B) Summary of differentially expressed transcription factor families. The column and pie chart showed the number and proportion of each transcription factor family, respectively. (C) Heatmap of expression pattern analysis of transcription factors. The data are calculated using $\log_{10}$ (FPKM). RNAi-EV induced hairy roots are set as CK.

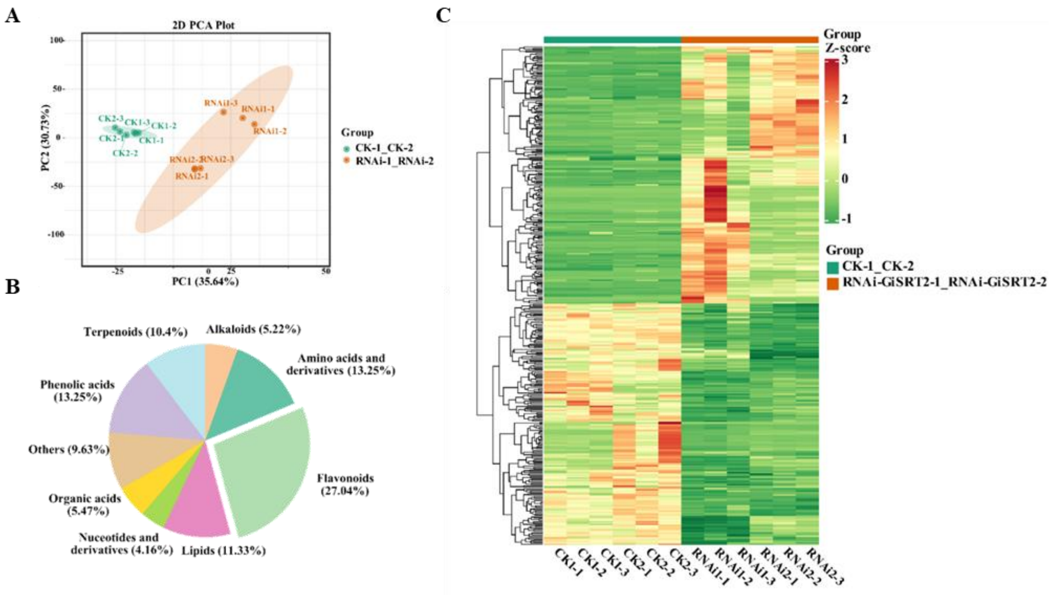

Figure S8. Analysis of the metabolites detected by the metabolome. (A) Principal component analysis of all the samples. (B) Ratio of different types of DAMs. (C) Clustered profiles of DAMs. The color scale on the right represents re-processed $\log_{10}$ (FPKM) using heatmap, the expression variance for each metabolite is indicated by different colors ranging from low (green) to high (red). RNAi-EV induced hairy roots are set as CK.

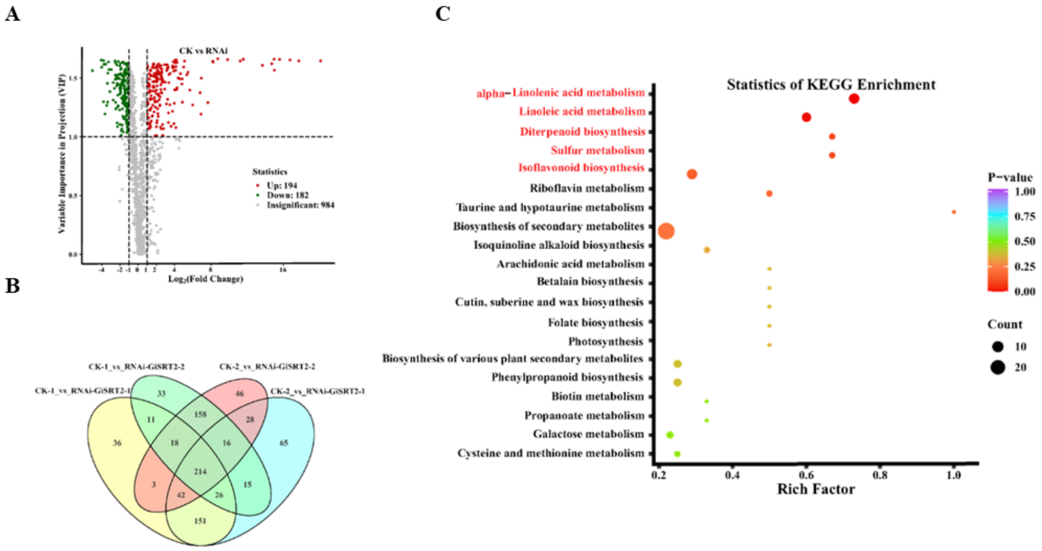

Figure S9. Overall analysis of the metabolomics data of RNAi-*GiSRT2*. (A) Volcano plots of the metabolic profiles in RNAi-*GiSRT2* and CK. (B) Statistics of the overlapped metabolites among the four comparison groups as indicated. (C) TOP 20 of KEGG enrichment in the differential metabolites. RNAi-EV induced hairy roots were used as the controls.

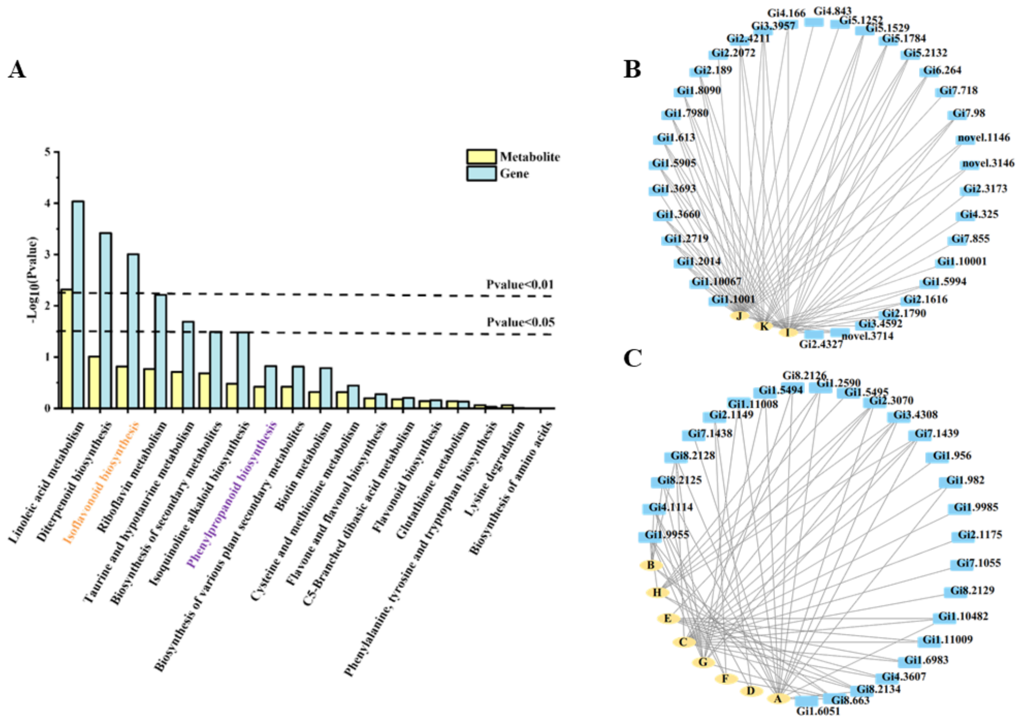

Figure S10. Correlation analysis between DAMs and DEGs. (A) KEGG pathway enrichment analysis of the DEGs and DAMs. (B) Correlation network between the genes and phenolic acids involved in the phenylpropanoid biosynthetic pathway. (C) Correlation network between the genes and isoflavonoids. Letter A, daidzein-7-*O*-glucoside; letter B, biochanin A-7-*O*-glucoside; letter C, glyceollin III; letter D, liquiritigenin; letter E, vestitol; letter F, daidzein; letter G, 2,6,7,4′-tetrahydroxyisoflavanone; letter H, (3R)-vestitol; letter I, ferulic acid; letter J, sinapoyl malate and letter K, coniferyl alcohol. Genes and metabolites with their Pearson’s correlation coefficient ≥ 0.99 and $P < 0.01$ are displayed here.

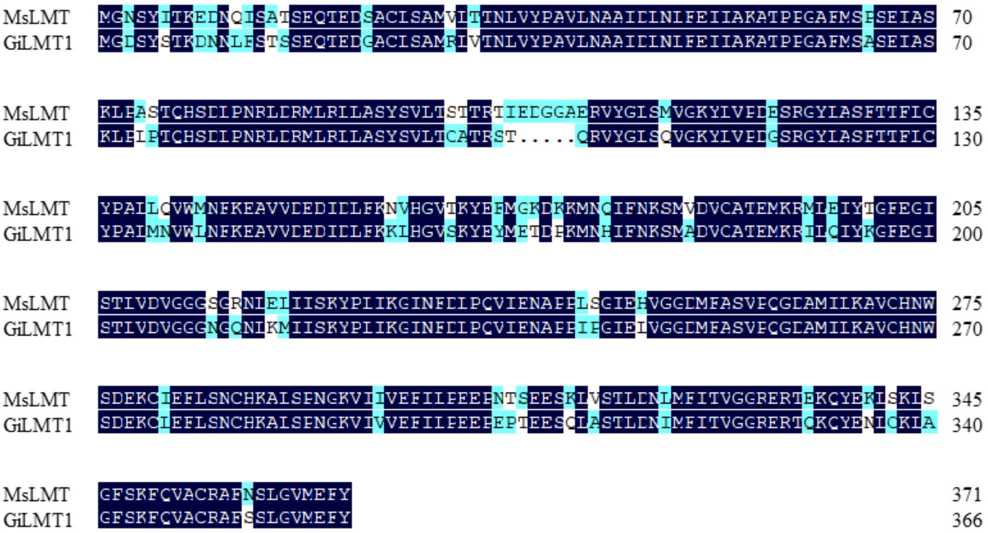

Figure S11. Amino acid sequence alignment of GiLMT1 and MsLMT. *MsLMT* (accession no. P93324) was cloned from *Medicago sativa* L. (Maxwell et al., 1993).

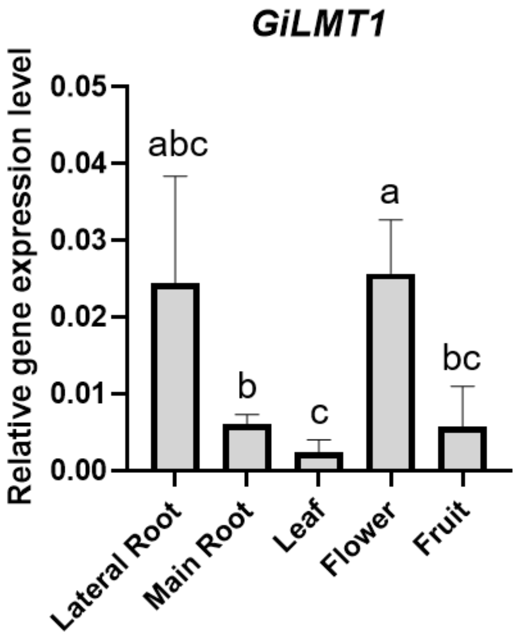

Figure S12. Tissues expression pattern of *GiLMT1* in *G. inflata*. The expression level of *GiLMT1* in lateral root, main root, leaf, flower and fruit of *G. inflata* were measured by qRT-PCR. *GiCOPS3* was used as the internal control. The different lower-case letter indicated significant difference at 0.05 level for the relative expression level among samples. Student's t-test, * $P < 0.05$, n = 3.

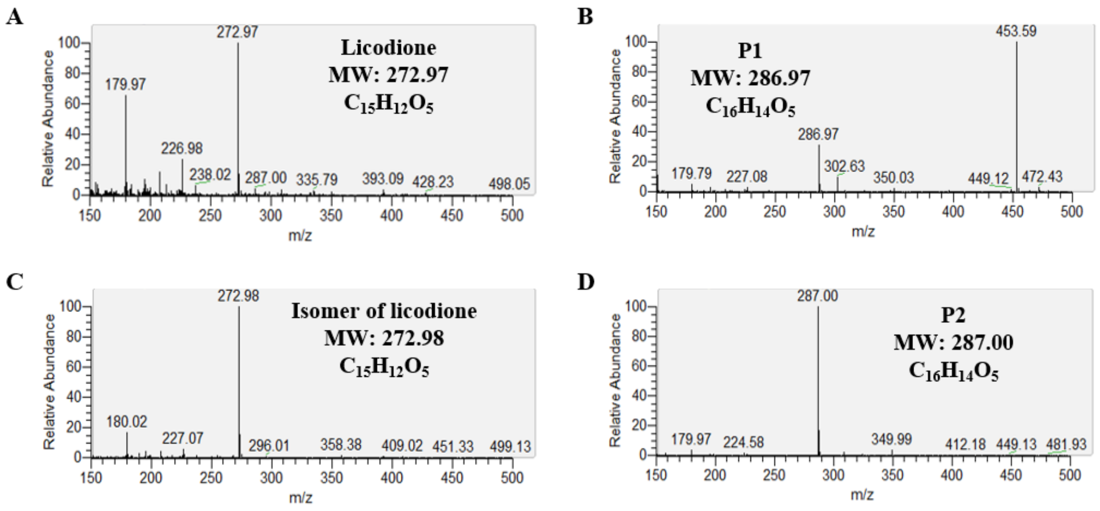

Figure S13. Identification of licodione, its isomer and their methylated products by mass spectrum. MS data of licodione (A), P1, the methylated product of licodione (B), isomer of licodione (C) and P2, the methylated product of licodione isomer (D) are as indicated. <to make the description a full sentence>

<Which peak (out of multiple peaks) in a panel represents the chemical you specified? Do you need an arrow to indicate? Or indicate the MW of the chemical (more straight forward than estimating from the chemical formula)?>
